# Adding Value to Goat Meat: Biochemical and Technological Characterization of Autochthonous Lactic Acid Bacteria to Achieve High-Quality Fermented Sausages

**DOI:** 10.3390/microorganisms5020026

**Published:** 2017-05-17

**Authors:** Miriam T. Nediani, Luis García, Lucila Saavedra, Sandra Martínez, Soledad López Alzogaray, Silvina Fadda

**Affiliations:** 1 Departamento de Ciencias de los Alimentos, Facultad de Agronomía y Agroindustrias, Universidad Nacional de Santiago del Estero. Av. Belgrano 1912, G4200 Santiago del Estero, Argentina; tnediani@gmail.com (M.T.N.); lgarcia@unse.edu.ar (L.G.); sandraluz08@gmail.com (S.M.); s.lopezalzogaray27@gmail.com (S.L.A.); 2 Centro de Referencia de BacteriasLácticas (CERELA-CONICET), Chacabuco 145, T4000ILC San Miguel de Tucumán, Argentina; lucila@cerela.org.ar

**Keywords:** goat meat, meat fermentation, fermented sausages, lactic acid bacteria, starter cultures, food quality, food safety

## Abstract

Quality and safety are important challenges in traditional fermented sausage technology. Consequently, the development of a tailored starter culture based on indigenous microbiota constitutes an interesting alternative. In the present study, spontaneously fermented goat meat sausages were created and analyzed using a physicochemical and microbiological approach. Thereafter 170 lactic acid bacteria (LAB) strains were isolated and preliminary characterized by phenotypic assays. The hygienic and technological properties, and growth and fermentative potential of isolates using a goat-meat-based culture medium were evaluated. All strains proved to have bioprotective features due to their acidogenic metabolism. Almost all grew optimally in meat environments. LAB isolates presented proteolytic activity against meat proteins and enriched amino acid contents of the goat-meat-based model. The most efficient strains were four different *Lactobacillus sakei* isolates, as identified by genotyping and RAPD analysis. *L. sakei* strains are proposed as optimal candidates to improve the production of fermented goat meat sausages, creating a new added-value fermented product.

## 1. Introduction

Goat meat represents an important part of food consumption and is one of the main components of several traditional dishes in the Mediterranean diet [1]. Historically, goat production in Argentina was associated with a subsistence economy and has been carried out by smallholders. This is the case of Santiago del Estero, located entirely in the semiarid Chaco region, where goats kept as livestock area very important resource generally linked to resource-poor farmers [2]. Generally, more tender and succulent young animals, denoted as ‘cabrito’ (kid goat), are offered for direct consumption while adult or discarded animals are not consumed; they are considered less tender and are associated with a more intense, undesirable, flavor and odor [3]. Consequently, better use of these meat sources is a major challenge in developing added-value goat meat products. In fact, the use of processes such as salting, smoking, and air-drying was the oldest way to preserve raw materials; however, its significance has recently increased as a way of transforming and diversifying meat products [4].

In order to improve the hygienic and sensory properties of fermented sausages, starter cultures are usually applied in industrial-scale processing. However, many artisanal products, strongly identified with a place of origin and traditions, do not apply starter cultures in their productions. Traditional dry sausages rely on natural contamination by environmental microbiota, a process that occurs during slaughtering and increases during manufacturing. Each processing unit has a specific “house microbiota” composed of useful microorganisms for fermentation and flavor development, but spoilage and pathogenic microorganisms are also present [5]. In fact, the safety of these artisanal products is somehow guaranteed by the addition of NaCl, nitrites/nitrates, the decreasing of water activity during the maturation, and the acidification produced by lactic acid bacteria (LAB), which constitute natural barriers to the development of pathogenic and/or contaminant microbiota [6]. Different species of LAB and Gram positive, coagulase-negative cocci (GCC) are the microorganisms primarily responsible for sausage fermentation [7]. LAB, in particular lactobacilli, contribute to the hygienic and sensory quality of fermented meat products mainly through their carbohydrate and protein catabolism, resulting in sugar depletion, pH reduction, the production of antimicrobial agents, and the generation of flavor compounds [8]. Accordingly, an efficient starter culture should be constituted of carefully selected strains with complementary technological properties and known safety features to guarantee uniformity in the production and improve the overall quality and safety of the end product. Proper selection of the starter culture involves a detailed study of the metabolic activity and technological properties of the species involved. Nowadays, another important challenge is to preserve the typical sensory quality of traditional sausages. Thus the development of a tailored starter culture based on indigenous bacteria can be a technological alternative, as these strains are better adapted to this environment and can better compete against natural microbiota [6]. On this basis, the aims of this study were: (i) to produce artisanal goat meat fermented sausages; (ii) to evaluate their quality and safety; and (iii) to isolate, identify, and characterize autochthonous *Lactobacillus* strains from the obtained fermented products. The hygienic and technological properties of isolates were evaluated in order to design an autochthonous starter culture capable of improving safety while preserving the typical sensory characteristic of traditional goat meat sausages produced in Santiago del Estero (northwest Argentina).

## 2. Materials and Methods

### 2.1. Preparation of Fermented Sausage and Sampling

The goat meat used in this study came from Creole x Boer castrated animals, provided by the Instituto Nacional de Tecnologia Agropecuaria—INTA (Santiago, del Estero, Argentina) with average live weights of 25 to 27 Kg. Wether goats were slaughtered at an age of 9–12 months, hand deboned, and chilled 24 h before processing. The artisan fermented sausage was prepared using two different formulations and following the methodology described by [9]. Fermented sausage formulations: A: 80% wether goat meat, 16% pork backfat, 2.8% NaCl, 0.75% dextrose, 0.75% sucrose, 0.2% white pepper, 0.02% nutmeg, 0.01% ascorbic acid, 1mg/kg sodium nitrite, 1 mg/kg sodium nitrate, and 0.01% polyphosphates. B: 40% wether goat meat, 40% pork meat, 16% pork back fat, 2.8% NaCl, 0.75% dextrose, 0.75% sucrose, 0.2% white pepper, 0.02% nutmeg, 0.01% ascorbic acid, 1mg/kg sodium nitrite, 1mg/kg sodium nitrate, and 0.01% polyphosphates. After being stuffed into pretreated bovine casings (42–46 mm diameter), the samples (15 cm; 150 g each sausage piece) were subjected to fermentation at 23–25 °C and 85 ± 5% relative humidity (RH) for three days. Further ripening was carried out at 12–15 °C, 70 ± 5% RH for 12 days. Fermentation and ripening were carried out in a laboratory ripening cabinet. Six replicates of each formulation (A and B) were carried out during one year.

For physical, chemical, and microbiological analysis, samples from A to B productions were taken after stuffing (t0; 0 h), during fermentation (t3; 3 days) and at the end of the maturation stage (t15; 15 days). All determinations were performed in triplicate.

### 2.2. Determination of Water Activity (a_w_) and pH

The pH values were obtained by directly inserting the tip of the probe MV-TEMP peachimeter (Digital Instruments, Taipei, Taiwan) into different portions of sausage samples. Water activity was determined on 5-mm sausage slices (45 mm diameter and 6.5 mm) using a ROTRONIC AwQuick carp instrument (New York, NY, USA) according to manufacturer specifications. Water activity and pH values, are expressed as mean values ± standard error (SE). Three independent measurements were performed on each sample.

### 2.3. Color Analysis

Color measurements were taken immediately after cutting the samples (to prevent color degradation as a result of light and oxygen) in accordance with the recommendations on color determination the American Meat Science Association [10]. Instrumental color analysis was performed using a Minolta spectrocolorimeter (Chroma meter CR-400, Osaka, Japan). L* (lightness), a* (redness) and b* (yellowness) values were determined by the CIE Lab system. Also Chroma (C*), which represents color saturation or purity, and Hue angle, defined as color wheel, with red–purple at angle 0° and 360°, yellow at 90°, bluish-green at 180° and blue at 270°,were calculated from a* and b* parameters. The colorimeter was calibrated before each set of measurements using a white tile. Ten measurements were taken in the core area and another 10 on the periphery of each sample and the results were averaged.

### 2.4. Microbiological Analysis and LAB Isolation

Samples were processed according to [11]: 25 g of each sausage were mixed with 225 mL of sterile 0.1% buffered peptone water (Britania, Buenos Aires, Argentina) and homogenized for 2 min in a stomacher machine (Lab blender 400, Seward Medical, London, UK). Appropriate decimal dilutions of the samples were prepared and plated in triplicate onto selective media to enumerate the microbiota. The following media and incubation conditions were used: (a) plate count agar (PCA, Britania) for total aerobic bacteria (37 °C; 48 h), (b) lauryl sulfate broth (Britania) coliforms by the technique of MPN (Most Probable Number) [12] (30°C, 48h) and confirmed in bile lactose broth (2%) brilliant green incubated for 24–48 h, (c) in EC broth (Britania) (45 °C, 48 h) for coliforms growing at 45 °C generally associated with enterohemorragic *E. coli*, (d) Baird Parker agar medium (BP; Britania) supplemented with egg yolk emulsion and kalium tellurite 20% [12] (37 °C; 72 h) for Coagulase-positive staphylococci (CPS), (e) H & L agar medium (Britania) for fungi and yeasts (28 °C; 7 days), (f) Pre-enrichment broths, Selenite Cysteine and bright green Tetrathionate (43 °C; 24 h)and Bismuth sulfite agar medium, bright green agar and Salmonella Shigella (SS) agar (35 °C; 48 h) [12]. were used for the determination of *Salmonella*. De Man Rogosa & Sharpe agar (MRS) (Britania) for LAB (30 °C; 48 h). For Gram positive Catalase positive Coagulase negative (GCC) Mannitol Salt Agar (MSA) (Britania) (30 °C; 48 h). Results are expressed as log CFU/g. After counting, means and standard error were calculated.

For LAB isolation purposes, attention was given to choose colonies from t3 and t15 samples from A and B sausage productions with different macroscopic morphology. Isolates were re-inoculated in MRS broth, incubated at 30 °C and checked for purity by streaking on MRS agar. Plates with pure cultures were used to test for cell morphology by phase contrast microscopy, Gram stain and catalase activity. Gram positive and catalase negative isolates were selected. These isolates were maintained as frozen stocks in MRS broth supplemented with 10% (v/v) glycerol at −20 °C during a month. Before experimental use, all LAB strains were recovered in MRS broth incubated at 30 °C and activated by twice inoculation in MRS broth.

### 2.5. Phenotypic and Technological Characterization of LAB

The Gram-positive, catalase-negative, no motile, non-spore forming, indole and nitrate negative isolates, were considered LAB and subjected to preliminary identification by traditional phenotypic methods: Growth at 15 and 45 °C in MRS broth; growth in MRS with 4% and 10% NaCl incubation at 30 °C for 48 h were determined as physiological traits [13]. The phenotypic characterization also included biochemical analysis such as: hydrolysis of esculin using esculin medium (0.6% peptone; 0.4% yeast extract; 0.4% tryptone; 0.1% glucose; 0.1% esculin; 0.01% Tween 80) [13]; hydrolysis of arginine using the Arginine Medium containing 0.3% l-arginine; 0.4% tryptone; 0.4% yeast extract; 0.1% glucose; 0.6% meat peptone; 0.01% Tween 80 [13]; ammonia production was detected using Nessler’s reagent (Merck); anaerobic production of gas from glucose and gluconate [13]. In addition fermentation profiles from carbohydrates (d-arabinose, l-arabinose, cellobiose, fructose, galactose, lactose, maltose, mannose, melibiose, melicitosa, raffinose, rhamnose, ribose, sucrose, salicin, trehalose and xylose) were the sugars assayed and polyalcohols (sorbitol and mannitol) [14]. The sugars and polyalcohols were filter-sterilized through a 0.22 mm syringe driven filter unit (Millipore, Saint-Quentin, France) and adjusted to a final concentration of 0.5% (*w*/*v*). Color changes of fermentation media were recorded at 24 and 48 h. Color change from red to yellow backlash was considered as a positive reaction.

### 2.6. Determination of Hygienic and Technological Properties of Fermented Sausage Isolates

#### 2.6.1. Decarboxylase Activity

The ability to produce biogenic amines by decarboxylation of the corresponding amino acid used as a precursor (l-histidine and l-tyrosine) was tested according to the method of [15]. The plates with the agar medium, supplemented with histidine (Anedra, Berlin, Germany) or tyrosine (Anedra) at 20 mg/L were spotted with the active strain and incubated anaerobically at 30 °C for 2–5 days. Growth of decarboxylating strains was easily recognizable because of their purple halo in the yellow medium.

#### 2.6.2. Screening for Antagonistic Activity

Detection of antagonistic activity in LAB strains was initially screened by means of an agar well diffusion assay (AWDA) [16]. BHI agar was used for *Listeria innocua*, used as indicator microorganism. Briefly, Petri dishes were overlaid with 15 mL of molten agar (1% agar), inoculated with 30 μL of an overnight culture of the indicator microorganism. Subsequently, 30 μL of the culture supernatant to be tested, heated to 100 °C for 3 min, to obtain sterile cell-free supernatant was seeded into the well. In addition, the overnight culture was neutralized with NaOH 3N, before being placed in the well, in order to discriminate antagonistic activity by the acid produced during growth. The plates were incubated aerobically for 24 h at 30 °C and subsequently examined for zones of inhibition. Inhibition was recorded as negative if no clear zone was observed around the agar well. Each antagonistic activity was related to the area (mm^2^) of the inhibition zone displayed. A positive control for antimicrobial activity, i.e., a bacteriocin-producing *Enterococcus mundtii* CRL35, was included.

#### 2.6.3. Lipolytic Activity

The lipolytic activity was determined on pork fat medium (PCA containing pork fat) [17]. The medium was distributed into plates and 10 µL of each culture were spread onto agar plates; incubated at 30 °C for 48 h. The appearance of a clear zone around the colonies was considered as an indicator for pork-fat lipolysis. The clear halos were measured (mm) from the edge of colony using a caliper. All tests were carried out in triplicate.

#### 2.6.4. Proteolytic Activity

Sarcoplasmic and myofibrillar proteins, extracted according to the method described by [18], were added at a concentration of 0.57 mg/mL to the agar medium containing 0.5% tryptone, 0.25% yeast extract, 0.1% glucose, 1.5% agar, pH 6.9. An overnight culture of each strain was centrifuged at 13,000× *g* for 5 min and the resulting pellet washed once with 20 mM phosphate buffer pH 7.0 and resuspended in an equal volume of the same buffer. Thirty microliters of cell suspension were applied into wells (6 mm diameter) bored in the agar plates. After incubation at 37 °C for 48 h, the agar layers were removed from Petri dishes and stained for 5 min in 0.05% (*w*/*v*) Brilliant Blue R (Anedra) in methanol:aceticacid:water (50:40:10) and distained in methanol:aceticacid:water (25:5:70). A clear zone surrounding the inoculated wells indicated proteolytic activity and its diameter measured in mm.

### 2.7. Growth, Adaption and Acidifying Ability in Meat Environments

#### 2.7.1. Meat Model System and Culture Conditions

Ten grams of goat semimembranosus muscle were homogenized with 100 mL of 20 mM phosphate buffer (pH 6.5) at 4 °C for 8 min in a Stomacher blender (Stomacher 400, London, UK). The homogenate was centrifuged (14,000× *g*, 20 min at 4 °C). The meat model system (MMS) consisted of the supernatant containing the sarcoplasmic proteins, which was filtered through Whatman paper, filter-sterilized through a 0.22 µm-pore-size filter (Steritop GP, Biopore, Buenos Aires, Argentina), and supplemented with 0.5% glucose and 0.01% Tween 80 according to [19]. The sterility of the system was confirmed by plating in PCA (37 °C; 48 h).

Each isolate was cultured in MRS over-night, cells were collected by centrifugation and the pellets resuspended in 20 mM phosphate buffer pH 7.0. This suspension was used to inoculate 10 mL of MMS at 2% and incubation at 30 °C for 96 h was carried out. The sampling was made at 0, 24, 48, 72, and 96 h for further analyses. Cell growth was analyzed by measuring cell viability by plating in MRS agar (30 °C; 48 h). Lactic acid and pH of the MMS inoculated with the isolate were determined at 0 and 24 h of incubation [20].

#### 2.7.2. Sodium Dodecyl Sulphate Polyacrylamide Gel Electrophoresis (SDS-PAGE)

The ability of the LAB isolates to hydrolyze sarcoplasmic proteins and peptides contained in the MMS was determined by electrophoresis under denaturing conditions (SDS-PAGE). The proteolytic activity was measured in samples taken at different times, 0, 24, 48, 72, and 96 h [18]. Un-inoculated MMS was used as control and incubated under the same conditions. SDS-PAGE was performed on 12% (w/v) polyacrylamide gels in vertical slab electrophoresis cells (BioRad Mini protean-3 System, Hercules, CA, USA) according to [18].

#### 2.7.3. Free Amino acid and Small Peptide Analysis

In order to analyze free amino acids and small peptides (aa) contents, non-inoculated MMS (to evaluate aa release by meat proteolytic enzymes) or MMS inoculated with the pre-selected isolates (for microbial peptidase activities and/or amino acid consumption) were analyzed. Free amino acids were measured according to the OPA spectrophotometric assay [21]. One ml of 12% trichloroacetic acid (TCA) was added to 0.5 mL of sarcoplasmic or myofibrillar extracts. After protein precipitation the extract was centrifuged (10,000× *g* for 10 min at 4 °C) and 50 μL supernatant aliquot was treated with o-phtaldialdehyde reagent. Results were expressed as absorbance at 340 nm. Amino acid concentration (mM) can be calculate from the following relationship: aa (mM)= ΔA340 F/ε, where ΔA340 is the experimentally observed change of absorbance at 340 nm using 1cm light path; F, dilution factor corresponding to the assay procedure; ε, molar absorption coefficient (6000 M^−1^·cm^−1^). All results are the mean of three replicate assays.

### 2.8. Genotypic Characterization of Isolates

Genomic DNA used as template was extracted according to [22]. Oligonucleotide primers (PLB16, 5′-AGAGTTTGATCCTGGCTCAG-3′; and MLB16, 5′-GGCTGCTGGCACGTAGTTAG-3′) were used to amplify the variable (V1) region of the 16S ribosomal RNA gene. The 500 bp fragment obtained of each isolate was purified and sequenced by the Sequencing Service of CCT-CONICET-Tucuman (San Miguel de Tucumán, Argentina). The sequences were analyzed using BLAST (The Basic Local Alignment Search Tool) from NCBI (http://www.ncbi.nlm.nih.gov/BLAST) and the Ribosomal Database (http://rdp.cme.msu.edu/).

### 2.9. Random Amplified Polymorphic DNA-PCR (RAPD-PCR) Analysis

TheDNA obtained from each isolate was used as template for RAPD-PCR reactions. Two different primers were singly employed: (i) M13 (5′GAG GGT GGC GGT TCT 3′) and (ii) XD9 (5′GAA GTC GTC C 3′) [23]. The following cycles conditions were used in the amplification experiments: (i) 40 cycles of [1 min/9°C, 20 s/45°C, 2 min/72°C]; (ii) 45 cycles of [1 min/94°C, 1min/36°C, 5 min/72 °C]. Amplification was performed in a Bioer XP cycler thermocycler (Bioer Technologies, Hangzhou, China). RAPD reactions were performed in a reaction volume of 25 μL containing 1.5 mM MgCl_2_, 2.5 μL Green Go Taq reaction buffer 5× (Promega, Biodynamics, Buenos Aires, Argentina), 1 μL of dNTP 5mM (Promega, Biodynamics), 1.25 μM of each primer, 1 U GoTaq^®^ DNA Polymerase (Promega, Biodynamics) and 1 μL of template DNA suspension or autoclaved water filtered with Milli-Q water purification system as the negative control sample. RAPD products were electrophoresed at 90 V on 2% agarose gel with 1× TAE buffer (0.04 M Tris–Acetate 0.001 M EDTA) and subsequently stained with a 0.1% Gel Red (Biotium, Fremont, CA, USA) solution.

### 2.10. Data Analysis

A completely randomized factorial design was used to analyze the effect of fixed factors (time and formulations) and their interactions. The comparison between different samples (experimental units) for each measured variable (microbiological, physicochemical parameters, etc.) was performed by factor analysis from ANOVA and Duncan’s test with a significance level of α < 0.05.

## 3. Results and Discussion

### 3.1. Physicochemical Analysis of Fermented Sausages

#### 3.1.1. pH and a_w_

The results of pH and a_w_ of fermented sausages are shown in Table 1. Significant differences over the time (*P* < 0.0001) were observed. As expected, at the onset of ripening (*t* = 0 days), pH and a_w_ values were not significantly different (*P* ≤ 0.05) between the A and B formulations being the initial values of pH = 6.14 and 6.07, respectively, while for both formulations a_w_ values were 0.99. During fermentation (*t* = 3 days), the pH and a_w_ decreased and significant differences (*P* ≤ 0.001) between the two formulations were observed. Important main effects of *Time* (***) and *Formulations* (**) were registered, although no significant interaction between formulations and time was observed, meaning that pH and a_w_, did not vary depending on the formulation and time (Table 1).The lowest pH (pH = 5.36) and water activity (a_w_ = 0.91) were obtained in sausages from A formulation during fermentation. The lower a_w_ value is consistent with the lower pH, as water loss is favored with decreasing pH [24]. Several authors observed a similar pH decrease during the fermentation step in European sausages [24,25]. The pH drop at the beginning of the fermentation is an essential requirement, since it contributes to the inhibition of undesirable microorganisms, accelerates the development of the typical curing red color, positively affecting the flavor, and reduces the water retention ability of proteins, ensuring the drying process [25]. At the end of ripening (*t* = 15 days), pH and a_w_ continued to decline. Statistical analysis showed that there were significant differences (*P* < 0.0001) between formulations. The lowest pH (pH = 5.10) at 15 days was observed in sausages from the A formulation (exclusively goat meat). The pH values achieved in both formulations were similar to fermented sausages from France, Spain, and Italy [26].Regarding a_w_ evolution, the lowest value was also achieved in sausages from formulation A (0.82); some authors found, at the end of maturation, a_w_ = 0.83 [27], while [28] found higher values in low-acid fermented sausages (a_w_ 0.93).

#### 3.1.2. Color

Color formation during ripening of sausages depends largely on pH and oxygen depletion, the content of nitrite/nitrate as well as the nitrate reductase activity of microbiota [29]. Changes involved in this process include decreasing the lightness (L*) as a result of drying, the increase in red green coordinate (redness) (a*) due to the formation of nitrosomyoglobin and decreasing values yellow blue coordinate (yellowness) (b*) [10]. Fermented sausages made with both formulations experienced the normal color development expected for traditional dry cured sausages. Detailed results for color parameters are depicted in Table 2. All variables, L*, a*, b*, chroma-saturation (C*) and Hue angle, tone (H*) were significantly affected by *Time* (*P* < 0.0001) and *Formulation* (*P* < 0.001 or *P* < 0.0001). Also, an important Time*Formulation interaction effect was obtained for a*, b*, C*, and H* (Table 3). This means that color coordinates were differently affected during the time according to sausage composition. At the initial time of ripening (t0), significant differences (*P*<0.05) between formulations were observed for L*, b*, and C* (Table 2). The lowest values of L* and b*, C* and H* were for sausages made exclusively with goat meat (formulation A). These values are consistent with the composition of the sausages, as it is known that goat meat is darker than pork meat due to a higher concentration of pigment [30]. On the third day, L*, b*, C*, and H* coordinates showed a significant decrease and the differences between formulations were significant (*P* < 0.05) (Table 2). Also, the pH experienced a sharp decrease at this time point, near to the isoelectric point of many meat proteins that has favored drying, provoking changes in the sausage surface and a decrease in brightness values. Regarding redness, significant variations were observed (*P* < 0.05) in both formulations, in line with the increment of GCC population (Table 3). Increases in redness during fermentation are ascribed to a typical reaction of color development in cured sausages, in which nitrosomyoglobin is produced and favored at a lower pH. Also, decreases in L*, b*, C*, and H* were observed, yielding less yellow, less brightness, less shade, and less luminous sausages with significant differences (*P* < 0.05) between formulations (Table 2). Drying produces a darkening of sausages due to the reduction of oxygen tension added to the increase of salt concentration, limiting myoglobin oxygenation and, in consequence, decreasing b* values [31,32]. The lowest values of L* were found for A sausages (51.86), in line with its lower pH (Table 1). Redness continued to rise significantly at 15 days of ripening in both formulations, being higher for sausages containing exclusively goat meat. Sausages made exclusively with goat meat (formulation A) had a higher intensity of red violet, with a higher blue component compared with inlays of formulation B. As mentioned before, goat meat has a darker color than beef and lamb, or pork and beef. In addition, the increase in a* values is related to the formation of nitrosomyoglobin [29]. In this sense, several authors found that the sausages showed a decrease in brightness (L*), yellowness (b*), saturation (C*), hue angle (H*), and increased redness (a*) after drying [29,33].

### 3.2. Microbial Analysis

The content of total microorganisms developing in fermented sausages along the ripening was analyzed by microbial count in selective cultures media (Table 3). Total mesophilic aerobic bacteria (TMA) counts ranged from 5.37 to 5.61 log CFU/g for B and A formulations, respectively, at the initial time of ripening, increasing their contents by approximately 2 log units at 15 days. There were no significant variations among formulations at t0 and t15 (*P* > 0.05). Fungi and yeast (H&L) counts remained constant and moderately low throughout the maturation period, with *Formulation* being the main effect producing the highest significant differences (Table 4). Several authors obtained similar results in spontaneously fermented sausages [34,35], while others observed higher counts (3–6 log CFU/g) [28,32,36]. Fungi and yeasts can have a positive effect on the formation of flavor during sausage ripening, although, if added during sausage manufacturing, they must be carefully selected [37,38].

#### 3.2.1. Microorganisms with Technological Importance

The evolution of LAB and GCC during the ripening of spontaneously fermented sausages made with the two different formulations (A, B) is depicted in Table 3. No significant difference (*P* > 0.05) was found for LAB and GCC contents between formulations, and no interactions between “Formulation*Time” were observed. At the beginning of the process (*t* = 0), LAB counts were between 3.28 to 3.33 log CFU/g for A and B, respectively. After three days (*t* = 3), significant increases for both microbial groups were observed in both formulations (3 to 5 log units increase). The highest counts were obtained in sausages from the A formulation, in accordance with the lower pH values found. Several authors reported similar results for LAB during fermentation [14,25,39]. At 15 days (*t* = 15), the final maturation stage, LAB counts between the two formulations were not significantly different (*P* > 0.05) (Table 3). Some authors found that LAB communities remained stable throughout the ripening process, while others reported that LAB had a slight decrease at the end of ripening due to the production of inhibitory compounds arising from the metabolism of sugars, lipids, and proteins and the depletion of carbon sources [28,29]. GCC in fermented sausages come from the surface of raw meat and human contact during processing [40], and its growth is promoted for its ability to survive to high salt concentrations, although they are sensible to acidic conditions [41]. Initial GCC counts (*t* = 0) ranged from 2.07 to 2.14 log CFU/g. Although some authors have reported higher initial contents of GCC in spontaneously fermented sausages (4.26 to 4.40 log CFU/g) [25,34,42]. After three days of fermentation (*t* = 3), a sharp increase in GCC population was observed in both formulations (4 log units increase) (Table 3). Several authors obtained similar results, with values ranging from 4–6 log CFU/g for this stage of fermentation [34,43]. At the final stage of ripening (*t* = 15 days), the GCC population remained almost stable, without significant differences between formulations during all ripening periods (0, 3, or 15 days). The microbial evolution observed confirms the findings of several authors who reported that LAB and GCC are microorganisms very well adapted to the harsh environmental conditions that develop during the production of dry cured sausages [23].

#### 3.2.2. Microorganisms Related to Food Spoilage and Food-Borne Illness

Among the non-desirable microbiota, minimal contents of coliforms at 45 °C and absence of *Salmonella* were registered in all sausages along the ripening period, indicating the adequate quality of the raw materials. Regarding coliforms at 30 °C, generally related to non-fecal enterobacteria, were registered at low contents (3 MPN/g); however, a peak (15 MPN/g) was observed on the third day of fermentation in sausages with the A formulation, although minimal levels were registered at the end of ripening (>3 NMP/g). *S. aureus*, which presented moderate content at the initial stages (3–5.3 log CFU/g), almost disappeared by the third day of fermentation, in line with pH reduction and the highest increase of LAB population, which favors the inhibition of pathogenic and spoilage microbiota [43]. When the contents of pathogen and spoilage microorganisms were analyzed by the Duncan test, significant differences, for almost all evaluated microorganisms, were observed by effect of *Formulations* and the interaction between “Formulation*Time” (*P* < 0.0001). This indicated that *Time* is an important factor that produces a negative effect on the growth of these microorganisms; *Formulations* also had an important effect, showing that A sausages presented, in general, a higher number of coliforms at 30 °C and a minor number of *S. aureus* as well as molds and yeast (Table 3). Also, an important interaction effect between “Time*Formulation” is observed, meaning that the observed changes were affected differently by the *Time* according to the *Formulation* involved.

### 3.3. Phenotypic Characterization of Lactic Acid Bacteria Isolated from Goat-Meat Fermented Sausages

The Gram-positive, catalase-negative, no motile, non-spore forming, indole and nitrate negative isolates derived from MRS agar plates were selected for morphological studies. From the 24 different fermented sausages [six replicates of each formulation; two processing steps (t3 and t15)], 170 isolates belonging to LAB were obtained and characterized by means of physiological studies using a set of 24 phenotypic tests. The majority of the isolated bacteria presented a rod-like morphology (150), while 20 strains corresponded to coconuts. This study was focused on rod-like bacteria. According to physiological and biochemical assays, the isolates were organized preliminarily in eight different *Lactobacillus* species-type groups. A different percentage of abundance from the total isolates was found in each cluster (Table 4). The dominant species-type groups, *L. sakei* (Group 1) and *L. curvatus* (Group 2), coincide with other works in which both species were reported as the most commonly found in European and Argentinean sausages [23,44]. All the isolates were able to grow at 15 °C and in the presence of 4% NaCl. This result is not surprising since for fermented sausages produced in the northwest of Argentina in artisanal facilities, salt concentration between 2.5% and 3.5% is usually applied. The ability to grow at 45 °C and in the presence of 10% salt was much less common. It is noteworthy that a low percentage of *L. sakei*-type isolates were able to produce NH_3_ from arginine; in general this species has this metabolic skill, which is important as a competitive advantage in meat [45]. On the contrary, all isolates clustered in Group 7 were able to metabolize this amino acid. In the same sense, a high percentage of isolates, with the exception of Group 4, showed an ability to metabolize ribose, one of the most important sugars present in meat [46]. The other sugars, in general, seemed to be adequate substrates for acid production with the exception of melezitose, raffinose, arabinose, mannitol, and maltose, metabolized by only a few species-type groups. The results presented herein are in agreement with those previously published on the identification and characterization of LAB in Italian [47], Spanish [48], French [49], and Greek [50] naturally fermented beef/pork sausages.

### 3.4. Hygienicand Technological Properties of LAB Isolated from Goat-Meat Fermented Sausages

#### 3.4.1. Decarboxylase Activity

Biogenic amines (BA) are natural components of many fermented and non-fermented foods of animal and vegetable origin and may represent a food poisoning hazard. The production of BA in meat and meat products, by enzymatic decarboxylation of specific amino acids, has often been related to lactic acid bacteria [51]. In the present work LAB isolates showed no decarboxylase activity against the analyzed amino acids (tyrosine, histidine, and lysine). This constitutes an interesting feature in view of the formulation of starter cultures ensuring the safety of the fermented product.

#### 3.4.2. Adaption to Meat Environments

All LAB strains were able to growth optimally in the meat model system (MMS) after 24 h at 30 °C, achieving increases higher than 3 log units (Table S1). A higher growth was observed in this meat system than in the laboratory medium routinely used for LAB (data not shown); this reflects a direct relationship with the specific LAB origin. These results agree with our previous findings [19], where optimal growth of different strains isolated from artisanal fermented sausages during growth in a similar meat model system is reported. *L. plantarum*-, *L. sakei*-, and *L. curvatus*-type isolates revealed that the highest acidifying potential is observed at the lowest pH values (3.72 ± 0.02, 3.91 ± 0.02 and 3.89 ± 0.05, respectively) and the highest acid production at 24 h (Table S1). Acidogenic metabolism is an essential feature of LAB that will guarantee meat fermentation and the hygienic quality of the product. In fact, all strains were able to inhibit *Listeria* cells due to acid production (data not shown). Moreover, lactic acid has other functions during meat fermentation such as its contribution to color development by means of the chemical reduction of nitrites to nitric oxide, the development of a typical acidic flavor, and promoting the drying process, which have a direct influence on the final texture of the product [52]. Thus, five strains with the best growth and acidogenic potential were selected for further assays. They belong to the *L. sakei*-(UL10; UL4); *L. curvatus*-(UL8; UL9), and *L. plantarum*-(UL12) type preliminary groups.

#### 3.4.3. Ability to Hydrolyze Fats and Proteins from Meat

##### Lipolytic Activity

LAB isolates showed no clear halos on agar plates supplemented with pork fat, suggesting the absence of lipolytic activity. This result is in accordance with [53], which reported the weak lipolytic system of lactic acid bacteria as well as very slight lipolytic activity in fermented meat products. However, it is known that LAB esterases are able to degrade esters, directly influencing the aroma of final products such as cheese or yogurt [54].

##### Proteolytic Activity

**Figure 1:**
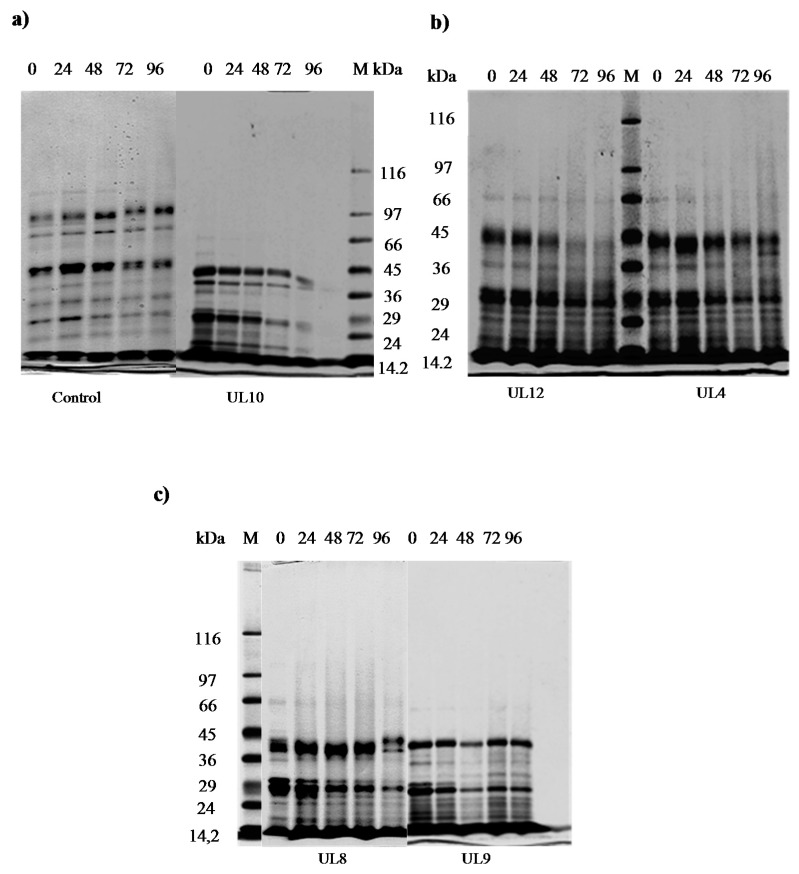
SDS-PAGE patterns of sarcoplasmic proteins present in the goat meat model system (MMS) inoculated with selected LAB strains, incubated at 30 °C for96 h. (**a**) Non-inoculated control; UL10 isolate; (**b**) UL12; UL4 isolates; (**c**) UL8; UL9 isolates. Lanes 1–5: 0–96 h. M: molecular weight markers; kDa: kilodaltons.

The proteolytic activity was firstly assessed by the agar diffusion method on 150 isolates. Results showed that none presented clear halos on the agar plates containing sarcoplasmic or myofibrillar proteins after Coomassie blue staining, this method indicating no evidence of meat protein degradation (data not shown). In addition, sarcoplasmic protein degradation by SDS-PAGE was assayed on the five selected isolates (UL10, UL4, UL8, UL9, and UL12). This protein type was selected as screening analysis since previous works reported sarcoplasmic proteins as the preferred substrate of hydrolysis for LAB from meat origin [8]. Results indicate different extent of sarcoplasmic protein degradation by the strains during 96 h of fermentation in the meat model system at 30 °C (Figure 1). The isolates UL12, UL10, and UL4 showed the most noticeable hydrolytic profiles at the end of fermentation (96 h), presenting a clear degradation of protein bands from 66 to 16 kDa. It is well known that muscle proteinases are primarily responsible for proteolysis, while bacterial enzymes become important during the later stages of ripening [8]. Bacterial contribution to meat protein degradation is especially due to their lactic acid production, which directly affects protein conformation and improves the activity of acidic meat proteases such as cathepsin D [47,55,56]. In fact, isolates UL10 and UL12, showing the highest protein degradation profiles, also presented the maximum acidifying ability (Table S1). On the other hand, slight changes in protein patterns were observed when UL8 and UL9 were tested.

##### Free Amino Acid and Small Peptide Analysis

LAB possess peptidases and aminopeptidases that contribute to the degradation of oligopeptides into small peptides and free amino acids, leading to an enrichment of these metabolites during ripening, which contributes to the sensory characteristics of the final product [57,58]. In the present work total amino acid and small peptides were analyzed during the fermentation of selected strains in MMS (Table 5). The method allows for quantifying the net content of amino acids, this being the result of amino acid release from the meat peptidase activity as well as the amino acid consumption and release by the microorganisms present in MMS. As recorded in SDS-PAGE profiles, amino acid analyses showed that UL10 and UL12 produced significant increases during fermentation (net change: 0.300 and 0.103, respectively) (Table 5) in line with previous works reporting that meat-borne LAB have a direct or indirect role in amino acid release during meat proteolysis [8]. In this sense, the adequate ability of UL10 and UL12 to produce free amino acids during meat fermentation could contribute to the sensorial quality of the final product.

### 3.5. Genotypic Characterization of LAB Strains Isolated from Goat-Meat Fermented Sausages with the Highest Technological Potential

**Figure 2:**
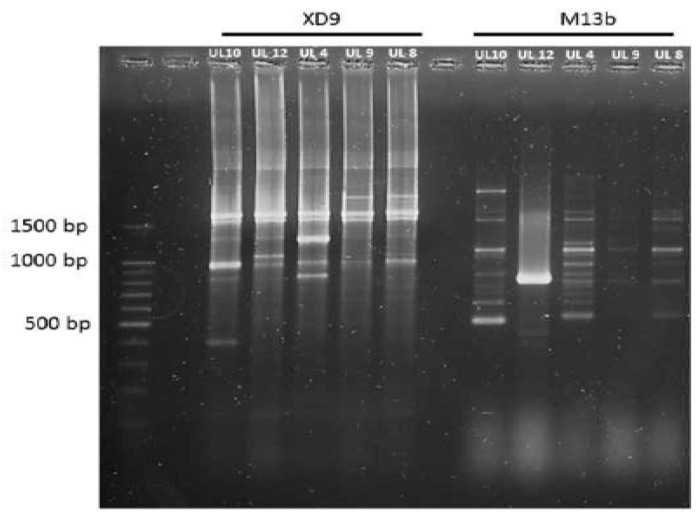
RAPD-PCR profiles obtained with XD9 and M13b primers representative of the selected LAB biotypes isolated from fermented goat sausages. M13b RAPD profiles: lane 1, Molecular weight marker (100bp DNA ladder); lane 3, biotype 1 (*Lactobacillus sakei* UL10); lane 4 biotype 2 (*Lactobacillus sakei* UL4); lane 5 biotype 3 (*Lactobacillus sakei* UL4); lane 6 and 7 biotype 4 (*Lactobacillus sakei* UL9 and UL8). XD9 RAPD profiles: lane 9 biotype 1 (*Lactobacillus sakei* UL10); lane 10 biotype 2 (*Lactobacillus sakei* UL4); lane 11 biotype 3 (*Lactobacillus sakei* UL4); lane 12 and 13 biotype 4 (*Lactobacillus sakei* UL9 and UL8).

The results from the molecular analyses of selected isolates by sequencing the variable region (V1) of the 16S rRNA gene revealed that even if the preliminary phenotypic characterization resulted in different species type groups, all the selected isolates belong to *Lactobacillus sakei*. In this sense, this species was already reported as better adapted to the fermented sausage environment [23,45,59]. Genotypic identification did not match perfectly in all cases with the results of biochemical methods, maybe due to the ambiguous responses of some isolates to certain assays. Conventional phenotyping methods have limitations for the identification of LAB species, and may make misidentifications [38,60,61]. While phenotypic assays are very important as a preliminary approach to unknown bacterial isolates, new molecular methods allow for overcoming the limitations of traditional methods. In addition, RAPD-PCR analyses, by using XD9 and M13b primers, showed the biodiversity among *L. sakei* isolates (Figure 2). Four dominant profiles were found for *L. sakei* isolates with either M13b or XD9 primers. The differences in the RAPD profiles of UL10, UL12, and UL4 showed they are different strains, while the similarities between UL8 and UL9 patterns indicate they are the same. It is noteworthy that four out the five selected isolates constitute different strains with high technological potential.

## 4. Conclusions

The quality and safety of fermented sausages made using two different percentages of goat meat revealed a high similarity, showing an appropriate physicochemical and microbiological quality of the final products. Only some color parameters presented significant differences between both formulations: the products made exclusively with goat meat had a darker color than those containing pork meat. On the other hand, this study provides a basis for the selection of native LAB strains for the production of goat-meat-based sausages. According to their competitiveness, the selected *L. sakei* strains, UL12 and UL10, were demonstrated to be good candidates for the formulation of an autochthonous starter culture. They possess desirable properties such as growth at 15 °C, resistance to moderate concentrations of NaCl, and fermentation of a wide variety of sugars such as ribose, present in meat. These isolates also demonstrated optimal growth and technological skills in meat environments such as acid production, meat protein degradation, and amino acid release, which could contribute to improving the quality and safety of fermented sausages. Moreover, the absence of his- and tyr-decarboxylase activities guarantees the absence of histamine and tiramine, and in consequence the appropriate hygienic potential. This work is among the few studies focused on the development of a starter culture for goat-meat-based sausages that could be an efficient alternative to improve the production process while preserving typicity and guaranteeing the quality and safety of the final product.Ongoing studies will evaluate the hygienic and sensory quality of goat meat fermented sausages madewith the selected LAB in combination with a selected GCC.

Finally, this work constitutes an important contribution for boosting regional meat industries by creating new added-value fermented products based on meat from culled goats and creating other choices for marketing.

## Figures and Tables

**Table 1:** Evolution of pH and a_w_ in spontaneously fermented sausages containing goat meat.

		pH		a_w_	
***Time* (days)**	***Formulation***	**A**	**B**	**A**	**B**
0		6.14 ± 0.07 ^A^	6.07 ± 0.07 ^A^	0.99 ± 0.01 ^C^	0.99 ± 0.01 ^C^
3		5.36 ± 0.07 ^B^	5.52 ± 0.07 ^C^	0.91 ± 0.01 ^A^	0.94 ± 0.01 ^B^
15		5.10 ± 0.07 ^D^	5.20 ± 0.07 ^E^	0.82 ± 0.01 ^D^	0.88 ± 0.01 ^E^

Test: Duncan α = 0.05 for factor interactions. Mean values ± statistical error. Means with common letter are not significantly different (*P* ≤ 0.05). A: 80% goat meat; B: 40% goat/40% pork meat.

**Table 2:** Analysis of the color parameters of spontaneously fermented sausages made with two different formulations.

*Time* (Days)	0		3		15	
*Formulation*	A	B	A	B	A	B
L*	67.23 ± 0.96 ^D^	71.38 ± 0.96 ^E^	61.91 ± 0.96 ^C^	68.52 ± 0.96 ^D^	51.86 ± 0.96 ^A^	56.22 ± 0.96 ^B^
a*	3.84 ± 0.25 ^A^	3.53 ± 0.25 ^A^	4.90 ± 0.25 ^B^	4.04 ± 0.25 ^A^	16.21 ± 0.25 ^D^	14.55 ± 0.25 ^C^
b*	14.51 ± 0.15 ^D^	16.42 ± 0.15 ^E^	12.67 ± 0.15 ^B^	13.75 ± 0.15 ^C^	8.86 ± 0.15 ^A^	8.69 ± 0.15 ^A^
C*	15.01 ± 0.36 ^C^	16.80 ± 0.36 ^D^	13.58 ± 0.36 ^A^	14.33 ± 0.57 ^B^	16.82 ± 0.36 ^E^	13.62 ± 0.36 ^D^
H*	75.21 ± 0.57 ^D^	77.88 ± 0.57 ^D^	68.45 ± 0.57 ^C^	73.64 ± 0.57 ^D^	28.18 ± 0.57 ^A^	34.60 ± 0.57 ^B^

Test: Duncan α = 0.05 for factor interactions (Formulation*Time). Mean values ± statistical error. Means with common letter are not significantly different (*P* ≤ 0.05); A: 80% goat meat, 0% pork meat; B: 40% goat meat/40% pork meat; L* (brightness, 0 to 100); a* (redness); b* (yellowness); C*: Chroma (saturation) and H* (Hue angle, tone) CIE 1976.

**Table 3:** Total microorganism counts in spontaneously goat meat fermented sausages at different times (0, 3, and 15 days).

*Time Formulation*/Microorganisms	0		3		15	
	A	B	A	B	A	B
LAB ^a^	3.37 ± 0.37 ^A^	3.48 ± 0.37 ^A^	7.53 ± 0.37 ^B^	7.58 ± 0.37 ^B^	7.06 ± 0.37 ^B^	6.96 ± 0.37 ^B^
GCC ^a^	2.08 ± 0.19 ^A^	2.09 ± 0.19 ^A^	6.26 ± 0.19 ^B^	6.09 ± 0.19 ^B^	6.09 ± 0.19 ^B^	5.88 ± 0.19 ^B^
TMA ^a^	5.61 ± 0.12 ^A^	5.37 ± 0.12 ^A^	5.46 ± 0.12 ^A^	6.60 ± 0.12 ^B^	7.29 ± 0.12 ^C^	7.24 ± 0.09 ^C^
Colif. 30 °C ^b^	4 ^A^	<3 ^B^	15 ^C^	<3 ^B^	<3 ^B^	<3 ^B^
Colif. 45 °C ^b^	<3 ^A^	<3 ^A^	<3 ^A^	<3 ^A^	<3 ^A^	<3 ^A^
*S. aureus* ^a^	3.00 ± 0.07 ^A^	5.30 ± 0.07 ^B^	<2 ^A^	<2 ^A^	<2 ^A^	<2 ^A^
H & L ^a^	2.29 ± 0.26 ^A^	3.12 ± 0.26 ^B^	2.40 ± 0.26 ^AB^	4.17 ± 0.26 ^C^	2.47 ± 0.26 ^AB^	2.57 ± 0.26 ^AB^
*Salmonella*/25 g ^a^	Absence	Absence	Absence	Absence	Absence	Absence

Test: Duncan α = 0.05. Mean values ± statistical error. Means with common letter are not significantly different (*P* ≤ 0.05); ^a^: Log (CFU/g); ^b^: NMP/g; LAB: Lactic Acid Bacteria; GCC: Gram-positive, coagulase-negative cocci; TMA: Total mesophilic aerobic bacteria. Colif. 30 °C: Coliforms at 30 °C. Colif. 45 °C: Coliforms at 45°; *S. aureus*: *Staphylococcus aureus*; H & L: Yeasts and molds.

**Table 4:** Phenotypic properties of LAB isolated from spontaneously fermented sausages made with goat meat.

Phenotypic Characterization								
Preliminary Identification/Phenotypic Tests	G1 *L. sa*	G2 *L. cu*	G3 *L. pla*	G4 *L. catol*	G5 *L. carha*	G6 *L. ali*	G7 *L. bre*	G8 *L. far*
Number of isolates	41 ^a^	40	19	14	8	10	10	8
Morphology	B	B	B	B	B	B	B	B
catalase activity	-	-	-	-	-	-	-	-
CO_2_ from glucose	-	-	-	-	-	-	+	-
CO_2_ from gluconate	+	+	+	13 ^b^	+	+	+	-
NH_3_ from arginine	+	-	-	-	-	+	-	+
Esculin hydrolysis	61	80	-	93	87	+	+	+
Growth with 4% NaCl	+	+	+	+	+	+	+	+
Growth with 10% NaCl	-	-	-	-	-	-	-	90
Growth at 15°C	+	+	+	+	+	+	+	+
Growth at 45°C	-	-	-	-	-	-	-	90
Acid production from:								
D (+)Fructose	+	+	+	+	+	+	+	+
L ramnose	-	-	-	-	+	-	-	-
Sucrose	+	80	90	-	+	+	+	+
Melezitose	-	-	90	-	87	-	-	-
Raffinose	-	-	60	-	-	-	+	-
Lactic acid production from:								
D (−) Arabinose	-	-	69	-	-	-	+	-
D (−) Lactose	19	10	69	-	90	+	+	-
D (−) Galactose	+	+	74	+	+	-	+	+
D (−) Mannose	90	+	80	+	+	90	80	+
D (−) Mannitol	-	-	84	-	+	+	-	+
D (−) Maltose	93	-	+	-	+	-	-	-
D (−) Ribose	90	+	90	7	+	+	+	+
D (−) Melibiose	+	-	95	-	12	-	90	-
D (−) Salicin	+	+	68	14	+	+	+	-
D (−) Glucose	+	+	+	-	+	+	-	+

Preliminary identification of species by phenotypic and biochemical analyses; G: Group; *L. sa*: *Lactobacillus sakei*; *L. cur*: *Lactobacillus curvatus*; *L. pla*: *Lactobacillus plantarum*; *L. catol*: *Lactobacillus casei subsp. tolerans*; *L. carrha*: *Lactobacillus casei subsp. rhamnosus*; *L. ali*: *Lactobacillus alimentarius*; *L. bre*: *Lactobacillus brevis*; *L. far*: *Lactobacillus farciminis*; B: rod-shaped ; ^b^: positive percentage for each test and for each isolate; + (positive for all strains); - (negative for all strains).

**Table 5:** Amino acid and small peptide content in MMS (goat meat model system) inoculated with selected LAB isolates and incubated at 30 °C for 96 h.

Time Strains	0 h	24 h	48 h	72 h	96 h	Net Change
Control *	0.261 ** ± 0.02	0.266 ± 0.02	0.257 ± 0.02	0.257 ± 0.03	0.260 ± 0.06	−0.001
UL4	0.263 ± 0.01	0.287 ± 0.01	0.287 ± 0.04	0.283 ± 0.04	0.284 ± 0.05	0.021
UL8	0.261 ± 0.03	0.266 ± 0.01	0.263 ± 0.01	0.279 ± 0.01	0.268 ± 0.02	0.007
UL9	0.262 ± 0.01	0.278 ± 0.03	0.281 ± 0.03	0.352 ± 0.03	0.264 ± 0.04	0.002
UL10	0.262 ± 0.02	0.277 ± 0.02	0.281 ± 0.03	0.328 ± 0.03	0.572 ± 0.07	0.300
UL12	0.262 ± 0.01	0.372 ± 0.02	0.425 ± 0.02	0.360 ± 0.01	0.365 ± 0.01	0.103

* Sterile MMS; ** (DO_340nm_).

## Supplementary Materials

The following are available online at www.mdpi.com/2076-2607/5/2/26/s1, Table S1: Growth and acidification ability of *Lactobacillus* isolates in the goat meat model system (MMS) at 30 °C after 24 h.
