# Supplementary material for: Adding Value to Goat Meat: Biochemical and Technological Characterization of Autochthonous Lactic Acid Bacteria to Achieve High-Quality Fermented Sausages
Source: Microorganisms. 2017 May 17;5(2):26. doi: 10.3390/microorganisms5020026 (PMC5488097; doi:10.3390/microorganisms5020026)
Supplement: Supplementary file 1 [file microorganisms-05-00026-s001.pdf]

Table S1. Growth and acidification ability of *Lactobacillus* isolates in the goat meat model system (MMS) at 30 °C after 24 h.

**Isolates**	**pH**		**Titratable acidity** [1]		**Viability** [2]
	t24	t24	t0	t24	net growth[3]
UL12	3.7±0.06 A	1.50±0.01	7.1	9.0 C	1.9 B
UL94	3.8±0.01 AB	1.38±0.01	7.0	8.0 ABC	1.0 AB
UL60	3.9±0.09 ABC	1.37±0.01	7.3	8.0 ABC	0.7 AB
UL57	3.9±0.19 ABC	1.50±0.01	7.3	8.5 ABC	1.2 AB
UL55	3.9±0.09 ABC	1.37±0.01	7.1	8.0 ABC	0.9 AB
UL54	3.9±0.13 ABC	1.36±0.01	7.1	8.0 ABC	0.9 AB
UL61	3.9±0.10 ABC	1.37±0.01	7.3	8.0 ABC	0.7 AB
UL123	3.9±0.14 ABC	1.37±0.02	7.1	8.0 ABC	0.9 AB
UL67	3.9±0.14 ABC	0.90±0.02	7.3	8.1 ABC	0.8 AB
UL64	3.9±0.10 ABC	1.50±0.01	7.3	8.5 ABC	1.2 AB
UL126	3.9±0.14 ABC	1.38±0.01	7.0	8.0 ABC	1.0 AB
UL49	3.9±0.12 ABC	1.50±0.01	7.3	8.5 ABC	1.2 AB
UL4	3.9±0.01 ABC	1.38±0.01	7.1	8.6 BC	1.5 AB
UL39	3.9±0.13 ABC	1.00±0.02	7.1	7.5 ABC	0.4 AB
UL34	3.9±0.06 ABC	1.45±0.01	7.0	8.1 ABC	1.1 AB
UL33	3.9±0.12 ABC	1.36±0.01	7.0	8.2 ABC	1.2 AB
UL32	3.9±0.12 ABC	1.36±0.01	7.0	8.2 ABC	1.2 AB
UL46	3.9±0.13 ABC	1.36±0.01	7.3	8.0 ABC	0.7 AB
UL45	3.9±0.14 ABC	1.35±0.01	7.3	8.2 ABC	0.9 AB
UL44	3.9±0.12 ABC	1.36±0.01	7.3	8.2 ABC	0.9 AB
UL41	3.9±0.12 ABC	1.36±0.01	7.3	8.2 ABC	0.9 AB
UL40	3.9±0.13 ABC	1.36±0.01	7.3	7.7 ABC	0.4 AB
UL84	3.9±0.06 ABC	1.38±0.01	7.1	8.0 ABC	0.9 AB
UL112	3.9±0.14 ABC	1.00±0.01	7.1	7.5 ABC	0.4 AB
UL11	3.9±0.05 ABC	1.36±0.01	7.1	8.6 BC	1.5v
UL106	3.9±0.08 ABC	1.00±0.01	7.0	7.5 ABC	0.5 AB
UL108	3.9±0.14 ABC	1.00±0.01	7.0	7.5 ABC	0.5 AB
UL92	3.9±0.06 ABC	1.38±0.01	7.0	8.0 ABC	1.0 AB
UL76	3.9±0.12 ABC	1.37±0.01	7.3	8.0 ABC	0.7 AB
UL96	3.9±0.10 ABC	1.38±0.01	7.1	8.0 ABC	0.9 AB
UL78	3.9±0.06 ABC	1.45±0.01	7.1	8.0 ABC	0.9 AB
UL10	3.9±0.14 ABC	1.38±0.01	7.0	8.0 ABC	1.0 AB
UL101	3.9±0.10 ABC	1.00±0.01	7.0	7.5 ABC	0.5 AB
UL25	3.9±0.12 ABC	1.36±0.01	7.0	8.0 ABC	1.0 AB
UL27	3.9±0.13 ABC	1.37±0.01	7.1	8.0 ABC	0.9 AB
UL20	3.9±0.13 ABC	1.35±0.01	7.2	8.2 ABC	1.0 AB
UL82	4.0±0.12 ABCD	1.26±0.01	7.0	8.1 ABC	1.1 AB
UL83	4.0±0.10 ABCD	1.26±0.01	7.1	8.1 ABC	1.0 AB
UL90	4.0±0.14 ABCD	0.8±0.01	7.0	7.5 ABC	0.5 AB
UL98	4.0±0.10 ABCD	1.3±0.01	7.0	8.1 ABC	1.0 AB
UL71	4.0±0.12 ABCD	0.8±0.01	7.1	7.9 ABC	0.8 AB
UL73	4.0±0.14 ABCD	1.4±0.01	7.1	8.1 ABC	1.0 AB
UL6	4.0±0.09 ABCD	1.0±0.02	7.2	7.8 ABC	0.6 AB
UL53	4.0±0.06 ABCD	1.26±0.01	7.1	8.1 ABC	1.0 AB

UL87	4.0±0.14 ^{ABCD}	1.4±0.01	7.0	8.1 ^{ABC}	1.1 ^{AB}
UL86	4.0±0.14 ^{ABCD}	1.4±0.01	7.0	8.1 ^{ABC}	1.1 ^{AB}
UL88	4.0±0.12 ^{ABCD}	1.26±0.01	7.1	8.2 ^{ABC}	1.1 ^{AB}
UL85	4.0±0.10 ^{ABCD}	1.26±0.01	7.1	8.1 ^{ABC}	1.0 ^{AB}
UL42	4.0±0.14 ^{ABCD}	1.26±0.01	7.1	8.1 ^{ABC}	1.0 ^{AB}
UL111	4.0±0.10 ^{ABCD}	1.3±0.01	7.1	8.1 ^{ABC}	1.0 ^{AB}
UL125	4.0±0.10 ^{ABCD}	1.26±0.01	7.1	8.1 ^{ABC}	1.0 ^{AB}
UL24	4.0±0.12 ^{ABCD}	1.0±0.02	7.0	8.0 ^{ABC}	1.0 ^{AB}
UL115	4.0±0.14 ^{ABCD}	1.26±0.01	7.0	8.1 ^{ABC}	1.1 ^{AB}
UL19	4.0±0.13 ^{ABCD}	1.3±0.01	7.1	8.1 ^{ABC}	1.0 ^{AB}
UL103	4.0±0.09 ^{ABCD}	1.26±0.05	7.0	8.1 ^{ABC}	1.1 ^{AB}
UL109	4.0±0.14 ^{ABCD}	1.26±0.01	7.1	8.1 ^{ABC}	1.0 ^{AB}
UL22	4.0±0.13 ^{ABCD}	1.0±0.02	7.0	7.9 ^{ABC}	0.9 ^{AB}
UL28	4.0±0.10 ^{ABCD}	1.0±0.02	7.0	8.1 ^{ABC}	1.1 ^{AB}
UL117	4.0±0.14 ^{ABCD}	1.0±0.01	7.0	7.6 ^{ABC}	0.6 ^{AB}
UL119	4.0±0.10 ^{ABCD}	1.3±0.01	7.0	8.1 ^{ABC}	1.1 ^{AB}
UL120	4.0±0.14 ^{ABCD}	1.26±0.01	7.1	8.1 ^{ABC}	1.0 ^{AB}
UL8	3.8±0.12 ^{ABCD}	1.38±0.01	7.0	8.0 ^{ABC}	1.0 ^{AB}
UL116	4.0±0.14 ^{ABCD}	1.26±0.01	7.0	8.1 ^{ABC}	1.1 ^{AB}
UL9	4.3±0.09 ^{ABCD}	0.9±0.02	7.1	7.8 ^{ABC}	0.7 ^{AB}
UL100	4.1±0.14 ^{ABCD}	1.0±0.01	7.0	7.6 ^{ABC}	0.6 ^{AB}
UL21	4.1±0.12 ^{ABCD}	1.0±0.02	7.0	7.8 ^{A}	0.8 ^{AB}
UL81	4.2±0.14 ^{ABCD}	1.4±0.01	7.0	8.1 ^{ABC}	1.1 ^{AB}
UL16	4.2±0.12 ^{ABCD}	0.7±0.01	7.1	8.0 ^{ABC}	0.9 ^{AB}
UL91	4.2±0.14 ^{ABCD}	0.9±0.01	7.0	8.5 ^{ABC}	1.5 ^{AB}
UL79	4.2±0.13 ^{ABCD}	0.9±0.02	7.0	7.5 ^{ABC}	0.5 ^{AB}
UL38	4.2±0.12 ^{ABCD}	0.7±0.01	7.1	6.9 ^{A}	–0.2 ^{A}
UL3	4.2±0.05 ^{ABCD}	0.9±0.01	7.1	7.5 ^{ABC}	0.4 ^{AB}
UL47	4.2±0.13 ^{ABCD}	0.9±0.02	7.3	8.1 ^{ABC}	0.8 ^{AB}
UL35	4.2±0.12 ^{ABCD}	0.8±0.01	6.9	7.9 ^{ABC}	1.0 ^{AB}
UL23	4.2±0.13 ^{ABCD}	0.9±0.02	7.0	7.9 ^{ABC}	0.9 ^{AB}
UL124	4.3±0.09 ^{ABCD}	0.9±0.01	7.0	7.9 ^{ABC}	0.9 ^{AB}
UL26	4.3±0.17 ^{ABCD}	0.9±0.02	7.0	7.9 ^{ABC}	0.9 ^{AB}
UL31	4.3±0.05 ^{ABCD}	0.9±0.02	7.0	7.5 ^{ABC}	0.5 ^{AB}
UL1	4.3±0.14 ^{ABCD}	0.9±0.02	7.3	7.9 ^{ABC}	0.6 ^{AB}
UL77	4.3±0.14 ^{ABCD}	1.4±0.01	7.3	8.1 ^{ABC}	0.8 ^{AB}
UL7	4.3±0.20 ^{ABCD}	0.9±0.02	7.1	8.1 ^{ABC}	1.0 ^{AB}
UL69	4.3±0.17 ^{ABCD}	1.4±0.01	7.3	8.0 ^{ABC}	0.7 ^{AB}
UL5	4.3±0.09 ^{ABCD}	0.8±0.02	7.0	7.8 ^{ABC}	0.8 ^{AB}
UL50	4.3±0.12 ^{ABCD}	1.0±0.02	7.1	7.6 ^{ABC}	0.5 ^{AB}
UL2	4.3±0.12 ^{ABCD}	0.9±0.02	7.2	7.8 ^{ABC}	0.6 ^{AB}
UL72	4.4±0.01 ^{ABCD}	0.8±0.01	7.3	7.8 ^{ABC}	0.5 ^{AB}
UL75	4.4±0.01 ^{ABCD}	0.8±0.01	7.1	7.7 ^{ABC}	0.6 ^{AB}
UL93	4.4±0.14 ^{ABCD}	0.8±0.01	7.0	7.7 ^{ABC}	0.7 ^{AB}
UL18	4.4±0.37 ^{ABCD}	0.8±0.02	7.0	7.7 ^{ABC}	0.7 ^{AB}
UL97	4.4±0.10 ^{ABCD}	0.8±0.01	7.0	7.8 ^{ABC}	0.8 ^{AB}
UL121	4.4±0.14 ^{ABCD}	0.8±0.01	7.1	6.9 ^{A}	–0.2 ^{A}
UL105	4.4±0.10 ^{ABCD}	0.8±0.01	7.1	7.9 ^{ABC}	0.8 ^{AB}
UL95	4.4±0.14 ^{ABCD}	0.8±0.01	7.0	7.9 ^{ABC}	0.9 ^{AB}
UL114	4.4±0.14 ^{ABCD}	0.8±0.01	7.0	6.9 ^{A}	–0.1 ^{A}

UL104	4.4±0.01 ABCD	0.8±0.01	7.1	7.9 ABC	0.8 AB
UL102	4.4±0.10 ABCD	0.8±0.01	7.0	7.2AB	0.2 AB
UL99	4.4±0.14 ABCD	0.8±0.01	7.0	6.9A	–0.1 A
UL70	4.5±0.37 ABCD	0.8±0.01	7.3	7.6 ABC	0.3 AB
UL17	4.5±0.13 ABCD	0.7±0.02	7.0	7.8 ABC	0.8 AB
UL107	4.6±0.01 ABCD	0.7±0.01	7.1	7.8 ABC	0.7 AB
UL14	4.6±0.12 ABCD	0.7±0.01	6.8	7.1 AB	0.3 AB
UL15	4.6±0.12 ABCD	0.7±0.01	6.9	7.8 ABC	0.9 AB
UL58	4.6±0.12 ABCD	0.8±0.01	7.0	7.7 ABC	0.7 AB
UL36	4.6±0.12 ABCD	0.7±0.01	6.8	7.1 AB	0.3 AB
UL68	4.6±0.08 ABCD	0.7±0.01	7.1	7.5 ABC	0.4 AB
UL66	4.6±0.08 ABCD	0.8±0.01	7.1	7.9 ABC	0.8 AB
UL13	4.7±0.12 ABCD	0.7±0.02	7.2	7.9 ABC	0.7 AB
UL56	4.7±0.12 ABCD	0.8±0.01	7.1	7.6 ABC	0.5 AB
UL63	4.7±0.12 ABCD	0.7±0.01	7.1	7.9 ABC	0.8 AB
UL43	4.7±0.15 ABCD	0.8±0.01	7.1	7.9 ABC	0.8 AB
UL80	4.7±0.12 ABCD	0.8±0.01	7.0	7.9 ABC	0.9 AB
UL89	4.7±0.12 ABCD	0.8±0.01	7.0	7.8 ABC	0.8 AB
UL65	4.8±0.12 BCD	0.7±0.01	7.1	7.9 ABC	0.8 AB
UL59	4.8±0.12 BCD	0.8±0.01	7.2	7.7 ABC	0.5 AB
UL30	4.9±0.37 CD	0.6±0.01	7.0	8.1 ABC	1.1 AB
UL37	4.9±0.37CD	0.6±0.01	6.8	7.4 ABC	0.6 AB
UL29	4.9±0.37CD	0.6±0.02	6.8	7.5 ABC	0.7 AB
UL52	4.9±0.37CD	0.5±0.01	7.1	7.6 ABC	0.5 AB
UL48	4.9±0.37CD	0.6±0.01	7.3	8.5 ABC	1.2 AB
UL62	5.0±0.37D	0.6±0.01	7.1	7.6 ABC	0.5 AB

Test: Duncan α = 0.05 for factor interactions. Mean values ± standard deviation. Means with common letter are not significantly different ($P \leq 0.05$). t0: 0 hours; t24: 24 hours. [1]: Titratable Acidity (% w / v lactic acid); [2]: Viability (log CFU/mL) and [3]: Net growth (log CFU/mL t_{24}–log CFU/mL t_0). Shading indicates that strains showed better growth and a greater decrease in pH occurred at 24 h in MMS.
